# Clinical efficacy of intraoral ultrasonography versus transgingival probing for measurement of gingival thickness in different gingival biotypes: a clinical trial

**DOI:** 10.1186/s13005-024-00422-4

**Published:** 2024-04-02

**Authors:** Maryam Alizad-Rahvar, Yaser Safi, Mahdi Kadkhodazadeh, Mohammad Parham Ghomashi

**Affiliations:** 1 Dept. Oral and Maxillofacial Radiology, School of Dentistry, Razi Herbal Medicines Research Center, Lorestan University of Medical Sciences, Khorramabad, Iran; 2 Dept. Oral and Maxillofacial Radiology, Shahid Beheshti University of Medical Sciences, Tehran, Iran; 3 Dental Research Center, Research Institute of Dental Sciences, Shahid Beheshti University of Medical Sciences, Tehran, Iran

**Keywords:** Ultrasonography, Gingiva, Clinical trial, Intraoral, Thickness

## Abstract

**Background:**

Transgingival probing is conventionally used for gingival thickness (GT) measurement. However, invasiveness is a major drawback of transgingival probing. Thus, researchers have been in search of alternative methods for measurement of GT. This study compared the clinical efficacy of intraoral ultrasonography and transgingival probing for measurement of GT in different biotypes.

**Materials and methods:**

This clinical trial was conducted on 34 patients requiring crown lengthening surgery. GT was measured at 40 points with 2- and 4-mm distances from the free gingival margin (FGM) of anterior and premolar teeth of both jaws in each patient by an intraoral ultrasound probe. For measurement of GT by the transgingival probing method, infiltration anesthesia was induced, and a #25 finger spreader (25 mm) was vertically inserted into the soft tissue until contacting bone. The inserted length was measured by a digital caliper with 0.01 mm accuracy. All measurements were made by an operator with high reliability under the supervision of a radiologist. Data were analyzed by t-test, Power and Effect Size formula, and intraclass correlation coefficient (ICC).

**Results:**

The two methods were significantly different in measurement of GT in both thick and thin biotypes at 2- and 4-mm distances (*P* < 0.001). The two methods had a significant difference in both the mandible (*P* < 0.001) and maxilla (*P* < 0.001) and in both the anterior (*P* < 0.003) and premolar (*P* < 0.003) regions. Although the difference was statistically significant in t-tests, the power and effect formula proved it to be clinically insignificant. Also, the ICC of the two methods revealed excellent agreement.

**Conclusion:**

The results showed optimal agreement of ultrasound and transgingival probing for measurement of GT.

**Trial registration:**

The study was approved by the ethics committee of Shahid Beheshti University of Medical Sciences on 2021-12-28 (IR.SBMU.DRC.REC.1400.138) and registered in the Iranian Registry of Clinical Trials on 2022-03-14 (IRCT20211229053566N1).

## Introduction

Periodontal phenotype is highly important in many dental procedures. Thus, several criteria have been defined for assessment of periodontal phenotype such as gingival thickness (GT), keratinized gingival width, and bone morphology. Determination of gingival biotype in different parts of the oral cavity plays a pivotal role in gingival health and prediction of periodontal response to different treatments [1]. Thus, measurement of GT and determination of gingival biotype are the first steps in many dental procedures such as periodontal surgery, orthodontic treatment, and implant placement [2]. Moreover, determination of gingival biotype and measurement of GT can help predict the risk of gingival recession and are also required for gingival tissue regenerative procedures following periodontal surgery [3]. They are also important in orthodontic treatment because the risk of gingival recession in the anterior mandible is higher in patients with a thin biotype, and it may necessitate a significant change in treatment plan (e.g., changing the expansion treatment plan to extraction). Furthermore, determination of gingival biotype and measurement of GT are imperative prior to orthodontic treatments that require mini-screw placement in the buccal mucosa in order to be able to estimate the primary stability of the mini-screws [4].

Several methods are available for measurement of GT. The qualitative methods for this purpose include visual inspection by the clinician and using a color-coded periodontal probe [5]. Transgingival probing is also a commonly used quantitative method for this purpose [5]. At present, transgingival probing is conventionally used for measurement of GT [6–8]. However, invasiveness is a major drawback of transgingival probing. Thus, researchers have been in search of alternative quantitative, non-invasive, and painless methods for measurement of GT. Cone-beam computed tomography (CBCT) and ultrasonography have been suggested for this purpose [2, 9]; however, ultrasonography is preferred due to its higher safety and no use of ionizing radiation. Accordingly, ultrasonography has been used for non-invasive measurement of GT [10]. Nonetheless, the literature is controversial regarding the accuracy of ultrasonography for measurement of GT, and significant differences have been reported between the values measured by ultrasonography in comparison with the transgingival probing method [7]. A previous study reported high measurement accuracy of ultrasonography and optimal reliability of its measurements; however, the clinical efficacy of this modality and the agreement between the measured and actual values are still questionable [8]. Another study reported that variations in the results may be due to not using an appropriate measurement tool [11]. However, some other studies reported high reliability of ultrasound measurements [10, 12, 13]. Another study even introduced ultrasonography as a non-invasive modality with a superior reliability than transgingival probing [14].

Variations exist in the anatomical points at which measurements have been made in the literature [11, 15]. Also, studies assessing the efficacy of ultrasonography for measurement of GT separately for each biotype (thick/thin) [5] or comparing ultrasonography and transgingival probing separately in the maxilla and mandible, or in the anterior and premolar regions of the jaws are lacking. Thus, this study aimed to compare the clinical efficacy of intraoral ultrasonography and transgingival probing for measurement of GT in different biotypes.

## Materials and methods

This study was conducted at the Oral and Maxillofacial Radiology Department of School of Dentistry, Shahid Beheshti University of Medical Sciences within a 6-month period in 2022. The study was approved by the ethics committee of the university (IR.SBMU.DRC.REC.1400.138) and registered in the Iranian Registry of Clinical Trials (IRCT20211229053566N1).

### Trial design

A clinical trial was conducted in which GT of patients was measured at 40 points first with the intraoral probe of an ultrasound and then by transgingival probing. The results were reported in accordance with the Consolidated Standards of Reporting Trials.

### Participants, eligibility criteria, and settings

The inclusion criteria were candidates with healthy periodontium and presence of all anterior and premolar teeth in the oral cavity. Due to the use of local anesthetic injection, it was crucial for ethical reasons that this study would have a gain for the patients thus candidates for crown lengthening surgery with a healthy gingiva were selected.

The exclusion criteria were tobacco use, intake of medications affecting gingival health such as phenytoin, pregnancy, presence of periodontal pockets, gingival inflammation, and gingival recession at the measurement sites.

The sample consisted of 34 patients including 17 eligible patients with a thick gingival biotype and 17 with a thin gingival biotype (as determined by visual inspection by a periodontist) that were selected after assessment of patient records and clinical periodontal examination of patients.

### Interventions

The records of patients presenting to the School of Dentistry of Shahid Beheshti University of Medical Sciences and a private radiology clinic in Tehran city who required crown lengthening surgery were assessed. Eligible patients underwent clinical periodontal examination, and after ensuring their gingival health and no inflammation or periodontal pockets around their anterior teeth and premolars, all teeth were probed at three points in the buccal surface using a UNC-15 periodontal probe (Premier Dental, USA). Written informed consent was obtained from all patients prior to their enrollment. All GT measurements were made by one operator with high reliability under the supervision of an experienced radiologist.

**Fig. 1:**
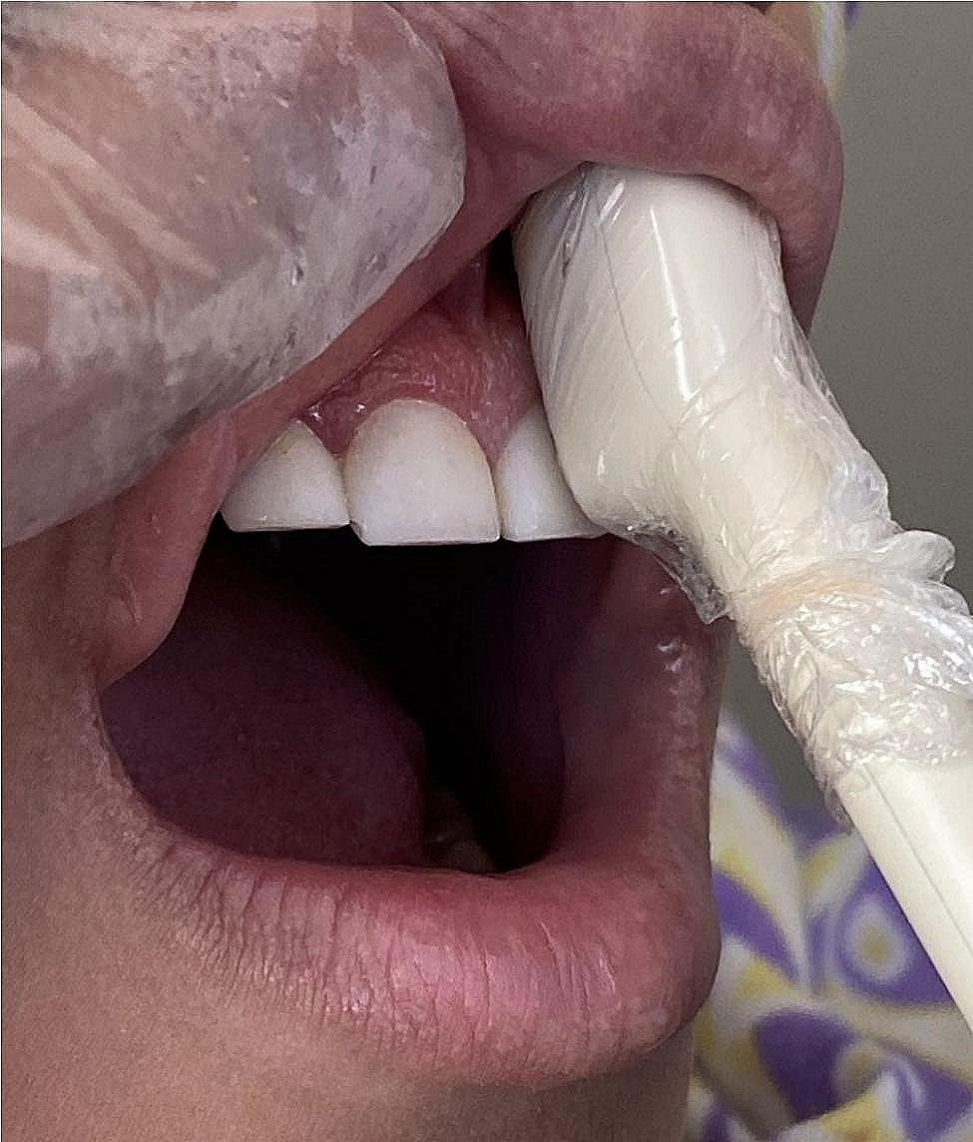
Positioning of intraoral probe of ultrasound for measurement of gingival thickness

For measurement of GT by ultrasonography, the patients were asked to have an upright position on dental chair, with their head in natural head position. The intraoral probe of ultrasound (B-Scan, E-CUBE 7, ALPINION, Korea) with 2 cm length and 12 MHz frequency was disinfected, and lubricant gel was applied over the probe detector as a conducting medium. The probe was positioned vertically at the mid-buccal region of anterior teeth and premolars of both jaws such that part of the probe was on the tooth and part of it was on the mid-buccal gingiva (Fig. 1). GT was measured at all 40 points by the measurement tool of ultrasound.

**Fig. 2:**
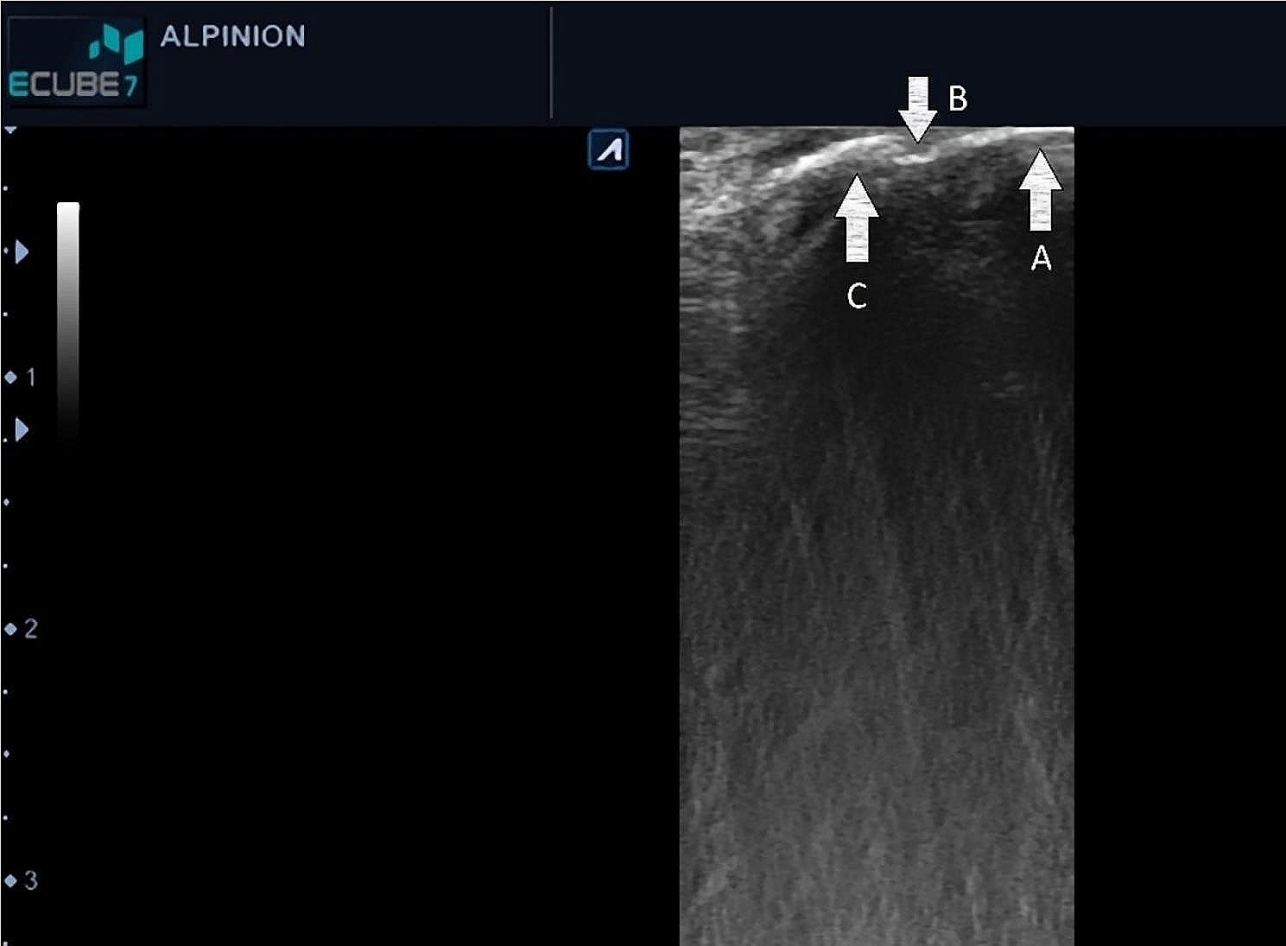
Real-time images showing the buccal surface of the tooth, cementoenamel junction, and buccal bone covering the root surface in the sagittal plane. In the right side, a hyperechoic area at the top (A-arrow) indicates the clinical tooth crown (buccal surface) on which, the probe is placed. The next hyperechoic line (C-arrow) indicates the buccal cortical plate. The intersection of the above mentioned two hyperechoic lines indicates the cementoenamel junction of the tooth (B-arrow). The entire hypoechoic tissue from the superior image margin (indicating the probe surface on the tooth and gingiva) to the hyperechoic area related to tooth or bone indicates the gingival thickness at the mid-buccal region

**Fig. 3:**
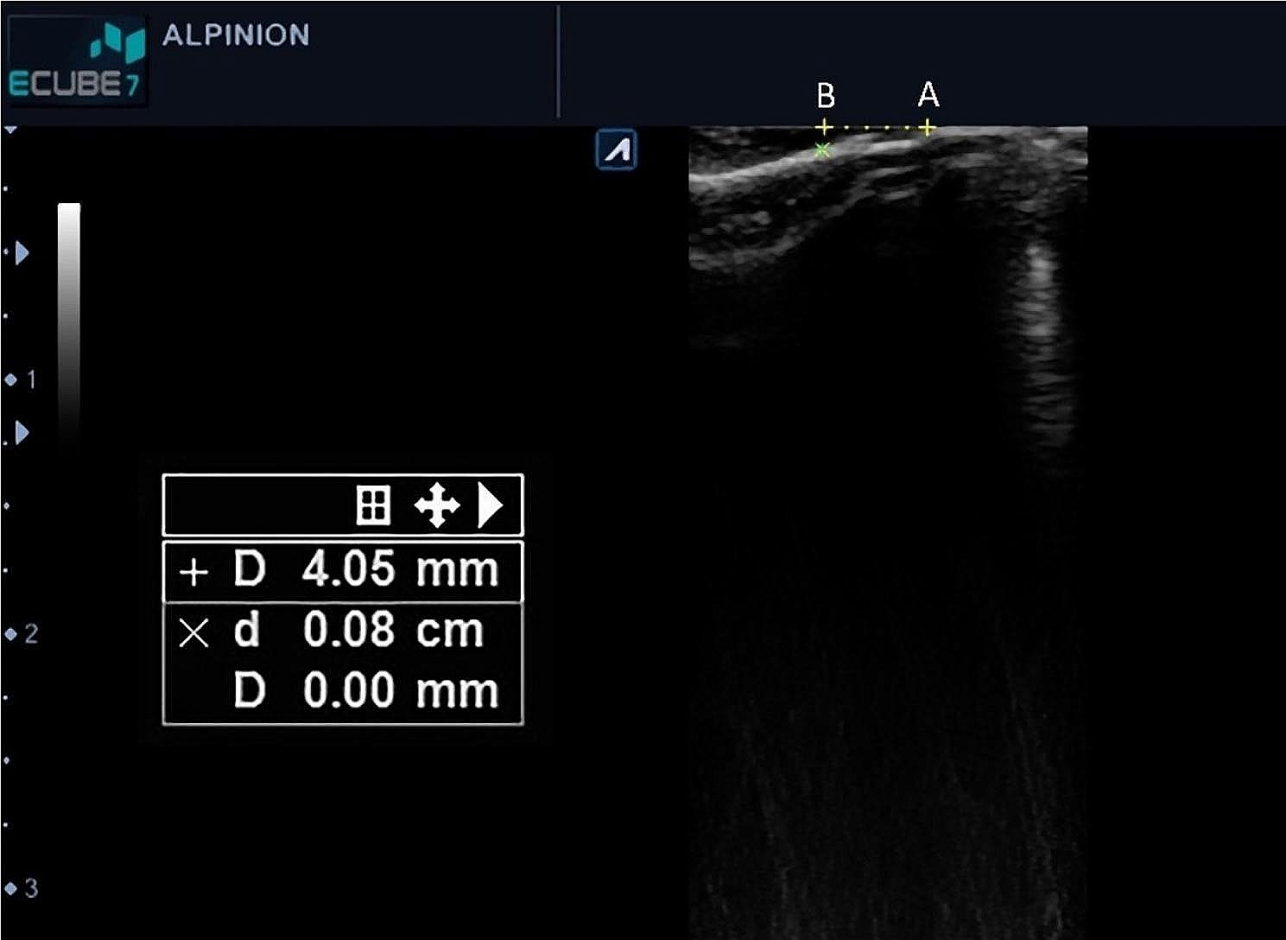
Measuring the gingival thickness (GT) at 4 mm distance from the free gingival margin. Points A and B indicate the gingival margin, and 4 mm distance from the free gingival margin, respectively. As shown, the GT at point B was 0.8 mm

Real-time images displayed on the monitor indicated the buccal surface of the tooth, cementoenamel junction, and buccal bone covering the root surface in the sagittal plane (Fig. 2). The entire hypoechoic tissue from the superior image margin (indicating the probe surface on the tooth and gingiva) to the hyperechoic area related to tooth or bone indicated the GT at the mid-buccal region. The measurement tool of the software was used to mark 2- and 4-mm distances from the free gingival margin (FGM), and then measure the GT from the superior margin of the image to the bone surface using the same measurement tool (Fig. 3). The measured values were recorded in Microsoft Excel 2019.

**Fig. 4:**
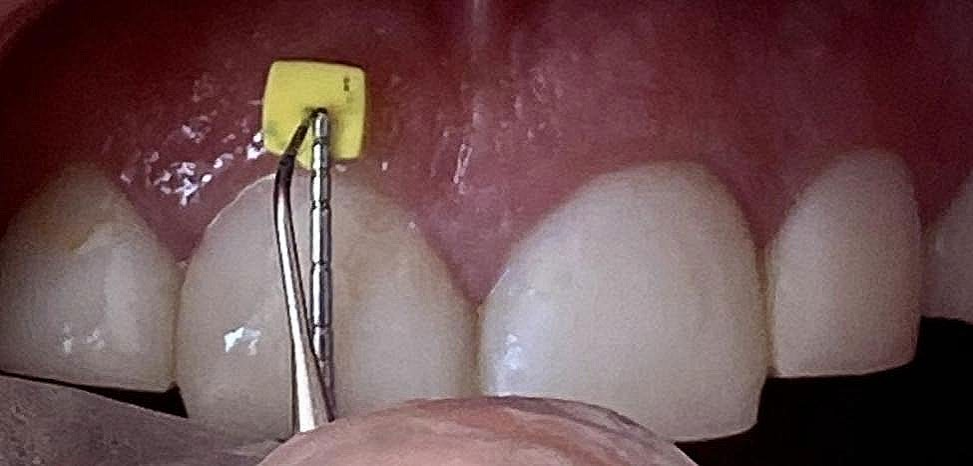
Marking a point at 2 mm distance from the free gingival margin at the mid-buccal of right central incisor by a periodontal probe and insertion of an endodontic spreader into the gingival tissue at this point for measurement of gingival thickness

For measurement of GT by the transgingival probing method, infiltration anesthesia was induced in the gingiva adjacent to the respective teeth by local injection of a few drops of 2% lidocaine plus 1:80,000 epinephrine (3% Citanest for patients with contraindications for lidocaine such as cardiovascular patients). After 10 min, a periodontal probe was used to mark 2- and 4-mm distances from the FGM at the mid-buccal region of the respective tooth (Fig. 4). Next, a #25 endodontic finger spreader (25 mm, 2% convergence; Mani, Japan) was vertically inserted into the gingival tissue until it reached the underlying bone for measurement of GT at the marked points. Upon contact with bone, the rubber stop of the spreader was adjusted and the spreader was removed. The length indicated by the rubber stop was measured by a digital caliper (Tech instruments, China) with 0.01 mm accuracy. The measured values were recorded in Microsoft Excel 2019.

After conventional measurement of the GT at 4 mm distance from the FGM, the patients were accordingly assigned to two groups of thick (> 1.5 mm) and thin (< 1.5 mm) gingival biotype [5].

### Outcomes (primary and secondary)

The main objective of this study was to compare GT measured by the intraoral probe of ultrasound with GT measured by transgingival probing. Comparison of GT measured by the two methods in the maxilla and mandible, anterior and premolar regions of the jaw, and at different levels from the FGM were the secondary outcomes.

### Sample size calculation

The sample size was calculated to be 17 according to a study by Savitha and Vandana [9] assuming α = 0.05, study power of 0.90, standard deviation of 0.25, and minimum significant difference of 0.2 between the two methods.

To increase the sample size compared with similar previous studies [9, 11], comparison of anterior and premolar regions of the jaws, and apico-coronal assessment of teeth, 40 points were assessed in each individual including two points at 2- and 4-mm distances from the FGM at the mid-buccal of the teeth from the left to the right second premolars in both the maxilla and mandible.

### Interim analyses and stopping guidelines

No interim analyses were performed, and no stopping guidelines were established.

### Randomization

Not applicable.

### Blinding

Blinding of the operator was not possible in the present study. However, the statistician who analyzed the data was blinded to the group allocation of participants.

### Statistical analysis

Data were analyzed by SPSS 26 (SPSS Inc., IL, USA). Paired t-test was used to compare the two methods, maxilla and mandible, and GT at 2- and 4-mm distances. Independent t-test was used to compare the values between the two jaws, anterior and premolar areas, and males and females. The intraclass correlation coefficient (ICC) was applied to assess the agreement of data for the mandibular right central incisor and second premolar, and right maxillary canine teeth between the two methods in each patient. Values < 0.4 indicated poor agreement, values between 0.4 and 0.59 indicated fair agreement, values between 0.6 and 0.75 indicated good agreement, and values > 0.75 indicated excellent agreement [8]. In addition to paired t-test, generalized estimating equation was also used for comparison of different areas in the same patient, which yielded results similar to t-test. To assess the clinical significance of the obtained difference between the two measurement methods, the formula of Power and Effect Size for independent means suggested by Kellar [16] was applied. Wherever significant differences were found between the measurements made by the two methods, the mean difference was divided by the standard deviation using the following formula and the obtained value was compared with the ranges reported by Kellar [16] (values up to 0.2 indicated very small difference, 0.2 to 0.5 indicated small difference, 0.5 to 0.8 indicated moderate difference, and 0.8 and higher indicated high difference) to find out the clinical significance of the difference between the ultrasonography and the transgingival probing measurements.

## Results

### Participant flow

**Fig. 5:**
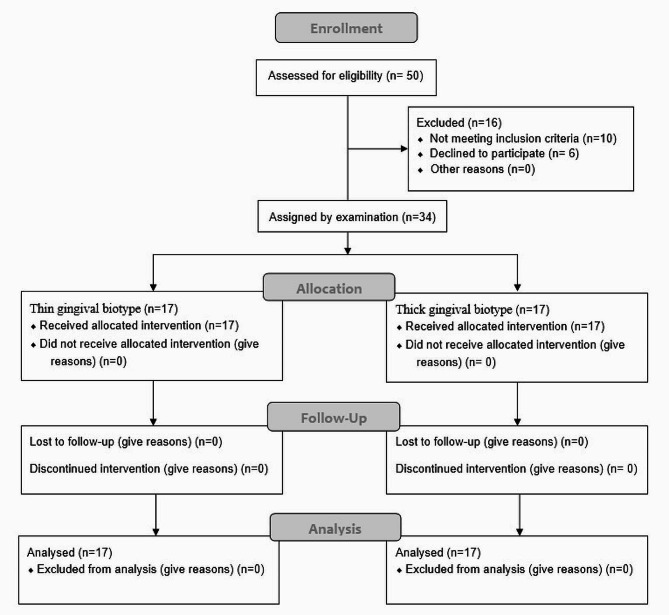
CONSORT flow-diagram of patient selection and allocation

The sample consisted of 34 patients including 15 females and 19 males between 18 and 35 years. GT was measured at 1360 points (40 sites in each patient) by both ultrasonography and transgingival probing. Figure 5 shows the CONSORT flow-diagram of patient selection and allocation.

### Harms

No patients were harmed during this study.

### Subgroup analyses

#### Primary outcome

Table 1 presents the measures of central dispersion for the measured GT by the two methods at 2- and 4-mm distances from the FGM. GT was measured at 2 mm distance by ultrasound at 680 points, at 2 mm distance by transgingival probing at 680 points, at 4 mm distance by ultrasound at 680 points, and at 4 mm distance by transgingival probing at 680 points. In general, the mean difference in the measured GT by the two methods was 0.2097 mm at 2 mm, and 0.2301 at 4 mm distance from the FGM. Comparison of GT measured by the two methods revealed significant differences at both 2 mm (*P* = 0.000) and 4 mm (*P* = 0.001) distances from the FGM.

**Table 1:** Measures of central dispersion for the measured GT (Gingival Thickness) by the two methods at 2- and 4-mm distances from the FGM (Free Gingival Margin)

Group analysis	Ultrasonography 2 mm	Transgingival probing 2 mm	Ultrasonography 4 mm	Transgingival probing 4 mm	Diff 2 mm	Diff 4 mm
Mean	1.3285	1.4349	1.4528	1.4177	0.2097	0.2301
Number	680	680	680	680	680	680
Standard deviation	0.23528	0.25966	0.28262	0.26035	0.15530	0.17505
Minimum	0.70	0.77	0.70	0.76	0.00	0.00
Maximum	2.20	2.23	2.50	2.38	1.10	1.20

Table 2 presents the measures of central dispersion for the measured GT by the two methods at 2- and 4-mm distances from the FGM in thin and thick biotypes. Comparison of the two methods for measurement of GT at 2 mm distance from the FGM in both thin (*P* = 0.000) and thick (*P* = 0.000) biotypes by paired t-test revealed significant differences. Comparison of the two methods for measurement of GT at 4 mm distance from the FGM in both thin (*P* = 0.000) and thick (*P* = 0.000) biotypes by paired t-test revealed significant differences as well.

**Table 2:** Measures of central dispersion for the measured GT (Gingival Thickness) by the two methods at 2- and 4-mm distances from the FGM (Free Gingival Margin) in thin and thick biotype groups

Gingival thickness		Ultrasonography 2 mm	Transgingival probing 2 mm	Ultrasonography 4 mm	Transgingival probing 4 mm	Diff 2 mm	Diff 4 mm	Ultrasonography 2 mm	Transgingival probing 2 mm
Thin biotype	Mean	1.2683	1.3531	1.3637	1.2397	0.1983	0.2330	1.2683	1.3531
	Number	394	394	394	394	394	394	394	394
	Standard deviation	0.20989	0.23372	0.26887	0.16160	0.15443	0.18941	0.20989	0.23372
	Minimum	0.70	0.77	0.70	0.76	0.00	0.00	0.70	0.77
	Maximum	2.10	2.22	2.50	1.49	0.82	1.20	2.10	2.22
Thick biotype	Mean	1.4115	1.5477	1.5755	1.6629	0.2255	0.2262	1.4115	1.5477
	Number	286	286	286	286	286	286	286	286
	Standard deviation	0.24331	0.25158	0.25401	0.14601	0.15539	0.15332	0.24331	0.25158
	Minimum	0.90	0.91	1.00	1.50	0.00	0.00	0.90	0.91
	Maximum	2.20	2.23	2.50	2.38	1.10	0.83	2.20	2.23

#### Secondary outcomes

Comparison of the two methods for measurement of GT at 2- and 4-mm distances from the FGM separately in the maxilla and mandible (Table 3) revealed significant differences at all points (*P* = 0.000) except at 4 mm distance in the maxilla (*P* = 0.860).

**Table 3:** Measures of central dispersion for the GT (Gingival Thickness) measured by the two methods in the maxilla and mandible

Jaw	Method	Mean	Number	Standard deviation	Std. Error Mean
Maxilla	Ultrasonography 2 mm	1.3703	340	0.24705	0.01340
	Transgingival probing 2 mm	1.4958	340	0.26020	0.01411
	Ultrasonography 4 mm	1.4594	340	0.30714	0.01666
	Transgingival probing 4 mm	1.4565	340	0.26024	0.01411
Mandible	Ultrasonography 2 mm	1.2868	340	0.21528	0.01167
	Transgingival probing 2 mm	1.3740	340	0.24474	0.01327
	Ultrasonography 4 mm	1.4462	340	0.25605	0.01389
	Transgingival probing 4 mm	1.3788	340	0.25496	0.01383

Comparison of the two methods for measurement of GT between the maxilla and mandible at 2- and 4-mm distances from the FGM (Table 4) revealed a significant difference only at 2 mm distance (*P* = 0.001) and not at 4 mm distance (*P* = 0.327).

**Table 4:** Mean difference between the maxilla and mandible at 2- and 4-mm distances from the FGM (Free Gingival Margin)

Difference in jaw	Number	Mean	Std. deviation	Std. Error of Mean
Maxilla at 2 mm	340	0.2297	0.16777	0.00910
Mandible at 2 mm	340	0.1898	0.13915	0.00755
Maxilla at 4 mm	340	0.2367	0.18555	0.01006
Mandible at 4 mm	340	0.2235	0.16389	0.00889

Comparison of the two methods in measurement of GT at 2- and 4-mm distances in the anterior and premolar regions (Table 5) revealed significant differences in all comparisons (*P* = 0.00) except at 4 mm distance in the premolar region (*P* = 0.165).

**Table 5:** Measures of central dispersion for the GT (Gingival Thickness) measured at 2- and 4-mm distances in the anterior and premolar regions measured by the two methods

Region	Method	Mean	Number	Std. deviation	Std. Error of Mean
Anterior	Ultrasonography 2 mm	1.2647	408	0.21929	0.01086
	Transgingival probing 2 mm	1.3884	408	0.24784	0.01227
	Ultrasonography 4 mm	1.3998	408	0.26290	0.01302
	Transgingival probing 4 mm	1.3581	408	0.24999	0.01238
Posterior	Ultrasonography 2 mm	1.4243	272	0.22615	0.01371
	Transgingival probing 2 mm	1.5046	272	0.26182	0.01588
	Ultrasonography 4 mm	1.5324	272	0.29281	0.01775
	Transgingival probing 4 mm	1.5071	272	0.25019	0.01517

Comparison of the two methods in measurement of GT in the anterior and premolar regions separately at 2- and 4-mm distances revealed no significant difference at 2 mm (*P* = 0.502) or 4 mm (*P* = 0.249) distance.

Comparison of the two methods in measurement of GT in males and females at 2- and 4-mm distances (Table 6) showed that the difference was not significant at 2 mm (*P* = 0.141) or 4 mm (*P* = 0.429).

**Table 6:** Measures of central dispersion for the measured GT (Gingival Thickness) at 2- and 4-mm distances in males and females by the two methods

Gender		Number	Mean	Std. deviation	Std. Error of Mean
Diff. at 2 mm	Male	380	0.2175	0.15839	0.00813
	Female	300	0.1999	0.15098	0.00872
Diff. at 4 mm	Male	380	0.2348	0.18155	0.00931
	Female	300	0.2241	0.16656	0.00962

#### ICC at three points for more accurate assessment

Mandibular right central incisor: The results revealed an ICC value of 0.754 (*P* = 0.000) for single measurement and 0.860 (*P* = 0.000) for the mean measurement at 2 mm distance, and ICC value of 0.678 for single (*P* = 0.000) and 0.808 (*P* = 0.000) for the mean measurement at 4 mm distance.

Maxillary right canine: The results revealed an ICC value of 0.630 (*P* = 0.000) for single measurement and 0.773 (*P* = 0.000) for the mean measurement at 2 mm distance, and ICC value of 0.456 for single (*P* = 0.003) and 0.627 (*P* = 0.003) for the mean measurement at 4 mm distance.

Mandibular right second premolar: The results revealed an ICC value of 0.775 (*P* = 0.000) for single measurement and 0.873 (*P* = 0.000) for the mean measurement at 2 mm distance, and ICC value of 0.662 for single (*P* = 0.000) and 0.797 (*P* = 0.000) for the mean measurement at 4 mm distance.

Power and effect test: Table 7 shows the results of power and effect test to determine the clinical significance of the differences found between the two methods in measurement of GT. As shown, the difference was small at all points and moderate at two points.

**Table 7:** Results of power and effect test to determine the clinical significance of the differences found between the two methods in measurement of GT (Gingival Thickness)

Comparison-magnitude of difference	0.2–0.5 Small	0.5–0.8 Moderate	0.8 High
Comparison of the two methods in measurement of GT in thin biotype at 2 mm distance	0.34		
Comparison of the two methods in measurement of GT in thick biotype at 2 mm distance		0.51	
Comparison of the two methods in measurement of GT in thin biotype at 4 mm distance	0.44		
Comparison of the two methods in measurement of GT in thick biotype at 4 mm distance	0.32		
Comparison of the two methods in measurement of GT at 2 mm	0.43		
Comparison of the two methods in measurement of GT at 4 mm	0.1		
Comparison of the two methods in measurement of GT in the maxilla at 2 mm	0.48		
Comparison of the two methods in measurement of GT in the mandible at 2 mm	0.38		
Comparison of the two methods in measurement of GT in the mandible at 4 mm	0.23		
Comparison of the two methods in measurement of GT in the anterior region at 2 mm		0.54	
Comparison of the two methods in measurement of GT in the anterior region at 4 mm	0.14		
Comparison of the two methods in measurement of GT in the premolar region at 2 mm	0.32		

## Discussion

This study compared the clinical efficacy of intraoral ultrasonography and transgingival probing for measurement of GT in different biotypes. The 12 MHz frequency was used in the present study due to its optimal penetration depth and resolution [17]. Initial hypothesis was that the Ultrasound method would be as accurate as the transgingival probing method. The results showed optimal agreement of ultrasound and transgingival probing for measurement of GT.

Although CBCT is a reliable method for measurement of GT [2], it should be noted that ultrasonography is superior to CBCT since it does not expose the patients to X-ray radiation. Also, CBCT shows minimal soft tissue details, has scatter radiation and limited field of view, and can have beam hardening artifacts due to dental materials and implants [6]. Moreover, the accuracy of CBCT depends on a number of factors such as the type of CBCT scanner, voxel size, image processing tools, and artifacts [7].

A recent systematic review conducted by Wang et al. [18] found no significant difference between CBCT and transgingival probing in measurement of GT and showed that they had a moderate agreement. However, they found a significant difference between ultrasonography and transgingival probing and concluded that ultrasonography was reliable only in the anterior region. Sönmez et al. [6] found no significant difference between ultrasonography and transgingival probing and concluded that they had optimal agreement.

The transgingival probing method has been performed differently in the available literature which may explain the variations in the reported results. Savitha and Vandana [9] used a graded probe for measurement of GT and due to insufficient accuracy, the values were rounded to the upper or lower limit. Sharma et al. [11] discussed that the reported significant difference in the study by Savitha and Vandana [9] was probably due to rounding of numbers, and used a digital caliper in their own study for the measurements. Also, Kloukos et al. [8] reported that application of periodontal probe may not be suitable for transgingival probing due to its blunt tip. Sharma et al. [11] suggested the use of a modified caliper; however, it was only applicable for the anterior region due to difficult access to the posterior region. In the present study, an endodontic spreader and a digital caliper were used for transgingival probing, similar to the study by Furtak et al. [19].

The ultrasound used in the present study was B-scan type, unlike the study by Savitha and Vandana [9], and had a stronger frequency than the ultrasound used by Eger et al., [14] and Savitha and Vandana [9] which would result in better penetration of waves into the tissue.

In the present study, the GT measured by ultrasonography varied between 0.7 and 2.5 mm while the measured GT ranged from 0.76 to 2.38 by the transgingival probing method. According to Studer et al., [20] the reported range of GT is variable in different races. Also, GT in males was averagely greater than that in females in the present study, which was in line with the results of Sharma et al., [11] and Esfahrood et al. [5].

In the present study, the results revealed that the ultrasound and transgingival probing methods were significantly different in measurement of GT in both thick and thin biotypes at 2- and 4-mm distances. Comparison of the two methods, irrespective of gingival biotype, at 2- and 4-mm distances also indicated significant differences at both distances between the two methods. Although significant differences were found between the two methods in the majority of the measurements, a definite judgment still cannot be made regarding the efficacy of ultrasonography because a large sample size can result in statistical significance of small differences, although they may be clinically negligible [21]. Accordingly, despite the presence of significant differences between ultrasonography and transgingival probing, some authors consider ultrasonography as a reliable method applicable in the clinical setting [9]. For instance, Gkogkos et al. [7] found a significant difference between ultrasonography and transgingival probing in the anterior mandible but added that this difference was not clinically significant. Therefore, for more accurate assessment of clinical differences in the present study, the Power and Effect analysis was carried out. Comparison of the two methods irrespective of gingival biotype at 2- and 4-mm distances revealed that the clinical efficacy of ultrasonography was comparable to that of transgingival probing. Comparison of the two methods at 2-mm distance separately in each gingival biotype revealed a small difference in the thin biotype and small-moderate difference in the thick biotype, indicating comparable clinical efficacy of ultrasonography and transgingival probing. At 4-mm distance, the difference was small for both thick and thin biotypes, which confirmed the equal clinical efficacy of the two methods. Moreover, the efficacy of ultrasonography was found to be the same in males and females. Similar to previous studies [9, 12], the majority of the comparisons were performed by t-test in the present study. Assessment of the agreement of the two methods by calculation of ICC revealed excellent agreement according to Kloukos et al., [8] at 2-mm (86%) and also at 4 mm (80%) distance for mandibular right central incisor. Reduction in agreement at 4 mm distance is probably due to poor adaptation and incorrect angulation of intraoral probe tip with the gingiva, because by an increase in distance from the free gingival margin, the shape of the mandible and mobility of the mucosa would complicate correct positioning of the probe tip and result in angulated position of probe in this area [1]. Nonetheless, excellent agreement (80%) was still achieved in this region. The ICC value was 77% at 2-mm distance at the site of right maxillary canine tooth, indicating excellent agreement. Lower ICC value compared with the value obtained at the site of central incisor can be due to the location of canine tooth in the arch complicating correct positioning of the probe due to anatomical barriers [8]. The ICC value decreased to 62% at 4-mm distance (good agreement), due to poor adaptation of the probe tip as the result of bony prominence in this region [8]. The ICC value was 87% at 2-mm distance at the site of mandibular right second premolar, indicating excellent agreement. The ICC value decreased to 79% at 4 mm, still showing excellent agreement. This reduction was probably due to poor adaptation of the probe tip at the depth of the vestibule and more posterior position of the tooth [17]. It should be noted that optimal efficacy of ultrasonography for measurement of GT has a direct correlation with correct positioning of the intraoral probe tip and absence of anatomical barriers.

As mentioned earlier, measurement of GT by transgingival probing and modified caliper is invasive and requires local anesthesia. Noninvasiveness is the main superiority of ultrasonography to transgingival probing and modified caliper. Aside from its invasiveness and patient discomfort [6], the measurement accuracy of transgingival probing depends on the measurement tool and experience and skills of the operator and may not be always reliable [6, 11]. Although modified caliper may have fewer errors than transgingival probing, it has difficulty in accessing the posterior region. Thus, it appears that ultrasonography may be comparable or because of its noninvasiveness even superior to the conventional method for measurement of GT [12].

This study had some limitations. Use of intraoral probe at the molar region was almost impossible because of the probe head size but if smaller probes were made, it would be achievable. Also, optimal adaptation of the probe tip is difficult in cases with a shallow vestibule, exostosis [17], and tilted or rotated teeth, necessitating excessive pressure of the probe over the tissue [22]. Moreover, the probe tip cannot be well positioned at the lingual side [23]. Operator-related errors of measurements may also occur [8, 19]. Thus, in a previous study, first the operator performed an in vivo measurement on an animal or cadaver for the purpose of calibration [7]. In the present study, one operator made all the measurements under the supervision of a radiologist. Thus, the problems related to possibly poor inter-examiner agreement were eliminated. Furthermore, the number of measured areas increased to decrease bias and increase accuracy. Not assessing the intra-examiner agreement was a limitation of this study since the patients could not be recalled again, and patients would not consent to undergo transgingival probing again due to its invasive nature. Also, high cost of ultrasonography machine is another drawback that limits its widespread application.

## Conclusion

Within the limitations of this study, the results showed that despite the presence of statistically significant differences between the two methods in some areas, the differences were not clinically important, and ICC values confirmed a good agreement. Thus, the intraoral probe of ultrasonography may be used for measurement of GT in most areas as a reliable, non-invasive modality with no X-ray radiation. Nonetheless, it cannot be recommended as a definite alternative to transgingival probing due to the aforementioned limitations and high cost of the machine.

## Data Availability

The datasets used and analyzed during the current study are available from the corresponding author on reasonable request.
